# The ferroptosis inducing compounds RSL3 and ML162 are not direct inhibitors of GPX4 but of TXNRD1

**DOI:** 10.1016/j.redox.2023.102703

**Published:** 2023-04-17

**Authors:** Dorian M. Cheff, Chuying Huang, Karoline C. Scholzen, Radosveta Gencheva, Michael H. Ronzetti, Qing Cheng, Matthew D. Hall, Elias S.J. Arnér

**Affiliations:** a Division of Biochemistry, Department of Medical Biochemistry and Biophysics, Karolinska Institutet, SE-171 77, Stockholm, Sweden; b Early Translation Branch, National Center for Advancing Translational Sciences, National Institutes of Health, Rockville, MD, 20850, United States; c Department of Selenoprotein Research and the National Tumor Biology Laboratory, National Institute of Oncology, Budapest, Hungary

## Abstract

Ferroptosis is defined as cell death triggered by iron-dependent lipid peroxidation that is preventable by antioxidant compounds such as ferrostatin-1. Endogenous suppressors of ferroptosis include FSP-1 and the selenoprotein GPX4, the latter of which directly enzymatically reduces lipid hydroperoxides. Small molecules that trigger ferroptosis include RSL3, ML162, and ML210; these compounds are often used in studies of ferroptosis and are generally considered as GPX4 inhibitors. Here, we found that RSL3 and ML162 completely lack capacity of inhibiting the enzymatic activity of recombinant selenoprotein GPX4. Surprisingly, these compounds were instead found to be efficient inhibitors of another selenoprotein, TXNRD1. Other known inhibitors of TXNRD1, including auranofin, TRi-1 and TRi-2, are also efficient inducers of cell death but that cell death could not be suppressed with ferrostatin-1. Our results collectively suggest that prior studies using RSL3 and ML162 may need to be reevaluated in the context of ferroptosis with regards to additional enzyme targets and mechanisms of action that may be involved.

## 1 Introduction

Glutathione peroxidase 4 (GPX4) is a selenoprotein using glutathione (GSH) and a highly reactive catalytic selenocysteine (Sec) residue to efficiently reduce lipid hydroperoxides, being a substrate specificity that distinguishes it from other GPX isoenzymes [1,2]. Originally named Phospholipid Hydroperoxide Glutathione Peroxidase (PHGPX) because of this eponymous activity, GPX4 has emerged as a key suppressor of the iron-dependent lipid peroxide-triggered mode of cell death named ferroptosis [1,[3], [4], [5], [6], [7]].

In 2012, Brent Stockwell's group coined the term ferroptosis for a cell death induced by the compounds erastin and (1S,3R)RSL3 (“RSL3”), using the RSL abbreviation for RAS-selective lethal because toxicity triggered by the compound is specifically pronounced in tumor cells mutated in the RAS family of small GTPases [8]. Cells undergoing ferroptosis are characterized by an accumulation of membrane lipid hydroperoxides, increased intracellular oxidative stress, and mitochondrial shrinkage. Ferroptosis is distinct from other forms of cell death as cytotoxicity triggered by treatment with its inducers, such as RSL3 or erastin, cannot be rescued by inhibitors of apoptosis, necrosis, nor autophagy. However, ferroptosis can be prevented by treatment with iron chelators or lipid antioxidants, of which ferrostatin-1 (Fer-1) seems to be the most potent [8,9]. Erastin targets the cystine-glutamate antiporter system (x_c_^—^), thereby lowering intracellular cysteine levels and counteracting GSH biosynthesis. With a diminished pool of GSH, reduction of lipid peroxides by GPX4 becomes impaired and can thereby trigger ferroptosis [3]. Additional ferroptosis inducers (FINs) have since been identified. Class I FINs, like erastin, act by depleting GSH, class II and class III FINs are defined as inhibiting GPX4 activity via either directly inactivating the enzyme or lowering its expression, respectively, while class IV FINs promote iron overload [10].

Using affinity-based chemo-proteomics, RSL3 was identified as the original class II FIN [8]. Although RSL3 has since been considered a direct GPX4 inhibitor, Vučković et al. noted that purified GPX4 did not seem to be directly inhibited by RSL3 and suggested that the cytosolic adaptor protein, 14-3-3ε, was required for inhibitory activity of RSL3 towards GPX4 [11]. In that study, only reduced but not oxidized forms of 14-3-3ε facilitated inhibition of GPX4 by RSL3, purportedly by enabling the interaction of the chloroacetamide of RSL3 with the Sec residue of GPX4. Since that study also used addition of cytosolic proteins in lysate to the assay, which was found to facilitate RSL3 effects on GPX4, it is possible that additional cellular factors can mediate the observed activity [11]. Together with RSL3, ML162 (also known as DPI7, or CID 3689413) and ML210 (DPI10, or CID 49766530) were identified in the original screen of novel ferroptosis inducing compounds, implying GPX4 as the cytosolic target for inhibition also by these compounds. ML162 shares the chloroacetamide moiety with RSL3, while ML210 contains a nitroisoxazole group required for its ferroptosis inducing activity. Eaton et al. bolstered the GPX4-targeting hypothesis by proposing a cellular conversion of the nitroisoxazole moiety of ML210 to α-nitroketoxime and subsequent dehydration to a nitrile-oxide, which would form a metabolite that can bind the Sec of GPX4 and thus inhibit the enzyme [12].

Another cytosolic selenoprotein with potent and wide reducing activity acting in cells in parallel to GPX4 is thioredoxin reductase 1 (TXNRD1). TXNRD1 uses NADPH to reduce the active site disulfide of thioredoxin (TXN) and other thioredoxin-fold proteins. TXNRD1 is a crucial enzyme for many cellular functions due to the general importance of the thioredoxin system in support of antioxidant enzymes such as peroxiredoxins and methionine sulfoxide reductases, catalysis of ribonucleotide reductase required for *de novo* synthesis of deoxyribonucleotides, supported activity of protein tyrosine phosphatases suppressing tyrosine kinase phosphorylation cascades, regulation of several key transcription factors, and many other important cellular functions [[13], [14], [15], [16]]. Similar to GPX4, many types of cancer cells show a particular dependence on TXNRD1 for survival and the enzyme has thus been the focus for drug development projects aimed at identifying inhibitors of TXNRD1, hypothesizing that such compounds may have anticancer properties [14,[17], [18], [19]]. The most efficient and specific TXNRD1 inhibitors yet described, as judged from studies of the pure enzyme as well as extensive proteomics studies and evaluation in animal models, are the FDA-approved gold-containing compound auranofin (Ridaura®) and the experimental Thioredoxin Reductase inhibitor (TRi) compounds TRi-1 and TRi-2 [17,[20], [21], [22], [23]]. Auranofin was identified early as a highly potent, though non-specific, TXNRD1 inhibitor in studies with purified enzyme [24]. The TRi compounds were discovered in a high-throughput inhibitor screen based upon enzyme activity assays with recombinant TXNRD1 [17].

Selenoproteins have been technically challenging to express and purify in recombinant form due to their unique translation machineries incorporating a selenocysteine residue at a redefined UGA stop codon [25]. It is only during the last few years that methods were developed for production of recombinant selenoproteins carrying an internal Sec residue, such as GPX enzymes. In contrast, TXNRD enzymes that have their active site Sec residue close to the C-terminal end of the protein could be produced at high yields already more than 20 years ago [[26], [27], [28], [29]]. Because recombinant selenocysteine-containing GPX isoenzymes have not been available until now, much direct enzymatic and kinetic analyses have been previously hindered.

Here, we evaluate for the first time the efficiency of direct GPX4 inhibition by RSL3, ML162, and ML210 and compare this inhibition with that of other GPX isoenzymes, as well as the inhibition of TXNRD1 by auranofin, TRi-1 and TRi-2. We discovered that not only did RSL3, ML162, and ML210 completely lack inhibitory activity towards pure GPX4, but RSL3 and ML162 were instead found to be highly efficient TXNRD1 inhibitors.

## 2 Results

### 2.1 RSL3 and ML162 are not direct biochemical inhibitors GPX4, but of TXNRD1

**Fig. 1:**
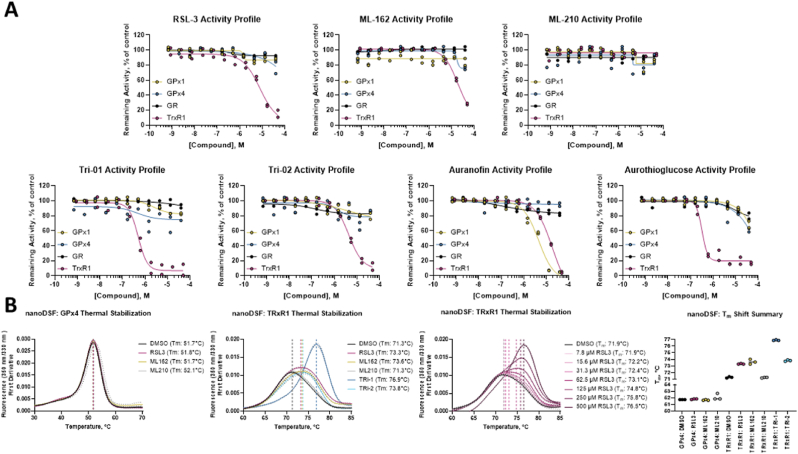
**Ferroptosis inducers do not inhibit pure GPX4 but are direct inhibitors of TXNRD1 *in vitro*.** A) Inhibitory activity of class I FINs, RSL3 (top left), ML162 (top middle), ML210 (top right), and known TXNRD1 inhibitors TRi-1 (bottom left), TRi-2 (bottom middle left), Auranofin (bottom middle right), and Aurothioglucose (bottom right) against GPX1, GPX4, GR, and TXNRD1 in activity assays using pure enzyme systems *in vitro*; Four-parameter dose-response curve fit to *n* = 2 technical replicates; B) nanoDSF experiments showing no effect on thermal stability of GPX4 by 100 μM RSL3, ML162, nor ML210 (left); Thermal stabilization of TXNRD1 by 100 μM TRi-1 and TRi-2, as well as RSL3, ML162, but not ML210 (middle left); Dose-dependent thermal stabilization of TXNRD1 by RSL3 (middle right); and a summary of triplicate thermal shifts with GPX4 and TXNRD1 in nanoDSF experiments. Data are presented as mean ± s.d. of *n* = 3 technical replicates.

We first set out to characterize inhibition of GPX4 by FINs in comparison to the inhibition of GPX1, reasoning that GPX4 inhibitors may also target other GPX isoenzymes. Using recombinant selenoproteins [2,[26], [27], [28]] and a selection of prior art ferroptosis inducing inhibitors [3] we were initially surprised to find that neither RSL3, ML162, nor ML210 inhibited either GPX isoenzyme (Fig. 1A, top panels). The only validated pan-inhibitors of GPX1 and GPX4 were mercuric chloride and mercaptosuccinic acid (MSA), which inhibited both isoenzymes with IC_50_ values under 10 μM in our assay conditions (Extended Data Fig. 1). We also assessed glutathione reductase (GR) and TXNRD1, discovering that RSL3 and ML162, but not ML210, inhibited TXNRD1, with IC_50_ values in these assays of 7.9 μM and 19.5 μM, respectively (Fig. 1A, top panels). TRi-1 and TRi-2 were confirmed as specific inhibitors of TXNRD1, with IC_50_ values under our assay conditions of approximately 500 nM for TRi-1 and 4.4 μM for TRi-2 (Fig. 1A, lower panels), in good agreement with our earlier findings [17]. Auranofin inhibited recombinant TXNRD1 with an IC_50_ of approximately 19.0 μM and showed additional promiscuity by exhibiting potent inhibition of GPX1 with an IC_50_ of 4.7 μM under these assay conditions; aurothioglucose only showed TXNRD1 inhibition, similarly to the TRi compounds (Fig. 1A, lower panels). The recombinant selenoproteins used here have high similarity with their native counterparts in terms of structure, enzymatic activity, and substrate specificity, with the main difference being that, mostly due to Gln or Lys suppression, the recombinant proteins have a lower Sec contents [2,26]. However, as only the Sec containing proteins have enzymatic activity these recombinantly produced variants can facilitate the identification of their inhibitors, as was done here.

### 2.2 RSL3 and ML162 show thermal stabilization of pure TXNRD1 but not pure GPX4

We further confirmed direct inhibition of TXNRD1 by RSL3 and ML162, and the lack of GPX4 inhibition by these compounds, using orthogonal biochemical assays (Extended Data Results and Extended Data Fig. 2) and next assessed the effects of these compounds on the enzymes using nano differential scanning fluorimetry [30,31]. TXNRD1 displayed a concentration dependent thermal stabilization after incubation with RSL3 or ML162, but binding to GPX4 could not be detected. The GPX4 DMSO negative control had a midpoint of protein melting, or T_m_, of 51.7 °C, which was unaffected by the addition of 100 μM RSL3 (51.8 °C), ML162 (51.7 °C), or ML210 (52.1 °C) (Fig. 1B, left panel). TXNRD1 showed a significant shift in T_m_ from 71.3 °C in the DMSO control to 73.3 °C and 73.6 °C upon treatment with 100 μM of RSL3 or ML162, respectively, with also TRi-1 and TRi-2 but not ML210 displaying thermal stabilization of the enzyme (Fig. 1B, middle left and right panels). RSL3 clearly displayed a dose-dependent stabilization of TXNRD1 with T_m_ shifts of +0.5 °C, +2.9 °C, and +4.6 °C using treatment of 31.25 μM, 125 μM, and 500 μM RSL3 (Fig. 1B, middle right panel). The apparent thermal stabilization of TXNRD1 and the lack of any thermal shift for GPX4 by the different compounds agreed well with the inhibition profiles on these selenoprotein enzymes by the different compounds.

**Fig. 2:**
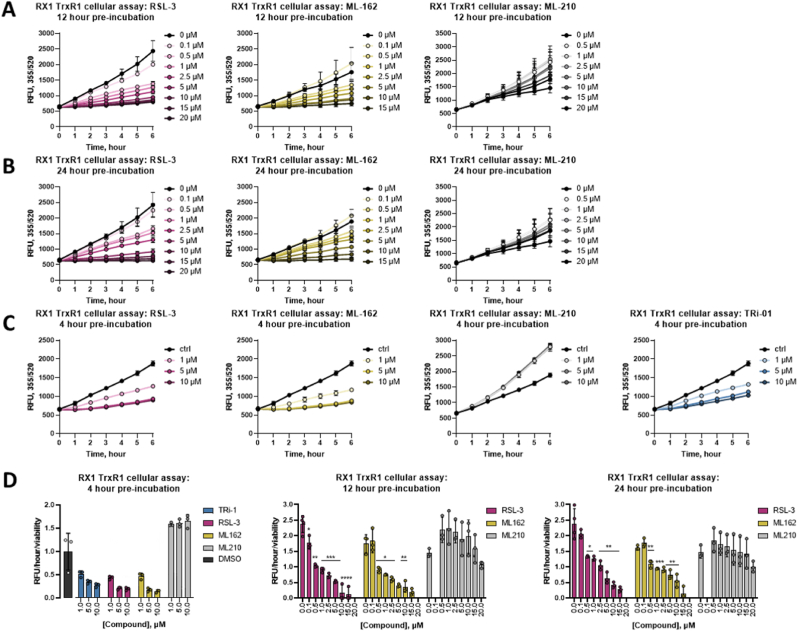
**Ferroptosis inducers inhibit TXNRD1 in cells.** A) Cellular inactivation of TXNRD1 measured with the RX1 activity probe at 12 h; B) cellular inactivation of TXNRD1 measured with RX1 activity probe at 24 h; C) cellular inactivation of TXNRD1 measured with RX1 activity probe at 4 h. Similar suppression in RX1 fluorescence signal was seen at all timepoints when comparing incubation of cells with TRi-1 (C, right panel), RSL3 (A-C, left panels), or ML162 (A-C, middle left panel), but not with ML210 (A-C, middle right panel), at several doses as indicated; D) summary of activity as controlled for viability is shown for 4 h (left panel), 12 h (middle panel), and 24 h (right panel) incubation times. Viability data is presented in **Extended Data**Fig. 3. Data are presented as mean ± s.d. of *n* = 3 biological replicates. Unpaired, two-tailed *t*-test; *P < 0.05, **P < 0.01, ***P < 0.001, ****P < 0.0001 in comparison to DMSO control; non-significant differences are not noted.

### 2.3 RSL3 and ML162 are inhibitors of cellular TXNRD1 activity

As the cellular context is crucial for the ferroptosis inducing activities of RSL3, ML162 and ML210, we next assessed whether these FINs could achieve TXNRD1 inhibition in cells. For this assessment we used the recently developed RX1 probe that specifically reports upon TXNRD1 enzymatic activity in live cells [32]. In A549 human lung cancer cells, maintained in growth medium supplemented with 100 nM sodium selenite for optimal selenoprotein saturation and TXNRD1 activity [33], a clear dose-dependent suppression in RX1 signal was seen with RSL3 and ML162 concentrations of 0.5 μM or higher, but not with ML210 (Fig. 2A and B). Interestingly, lower concentrations (0.5–1 μM) of ML210 showed a tendency to increase the TXNRD1 activity, while 20 μM ML210 led to slightly suppressed TXNRD1 activity (Fig. 2A and B, right panels). It should be noted that the effects of RSL3 and ML162 in concentration dependent suppression of cellular TXNRD1 activity also had a rather rapid onset, with 4 h of incubation with 1 μM or higher of either treatment with RSL3 or ML162 being sufficient for a suppression of the RX1 signal, thus showing similar efficacy to inhibition of TXNRD1 as TRi-1 (Fig. 2C). The increase in TXNRD1 activity seen using ML210 was also apparent after only 4 h of treatment (Fig. 2C**,** middle right panel). It should be noted that at this early time point and under these culture conditions no overt effects on viability was noted **(**Extended Data Fig. 3A**),** thus suggesting that TRi-1, RSL3 and ML162 effectively inhibit cellular TXNRD1 activity as an early event in their cellular mechanisms of action (and that ML210 if anything has an opposite effect). Later timepoints showed significant cell death upon treatment with RSL3 and ML162, but not ML210 (Extended Data Fig. 3B and C), thus the RX1 signals controlled for by cell viability are shown for all three timepoints (Fig. 2D).

**Fig. 3:**
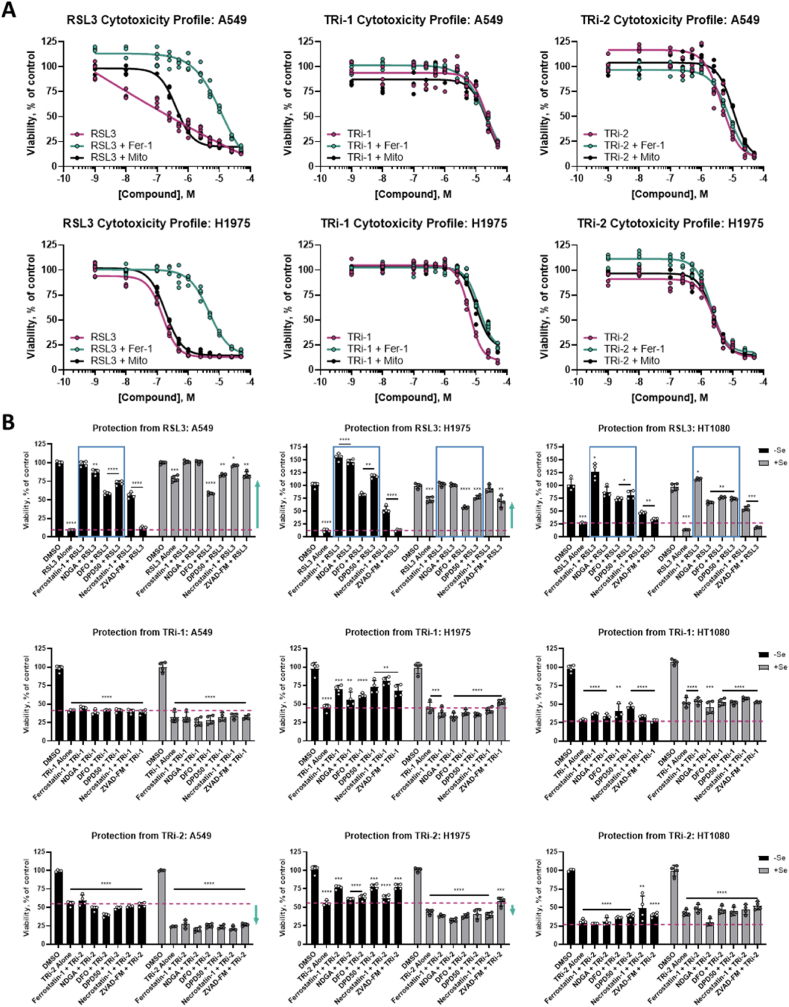
**RSL3, TRi-1, and TRi-2 have differential cellular cytotoxicity profiles.** A) Cytotoxic effects of RSL3 (pink curves, left panels), but not TRi-1 (pink curves, middle panels) or TRi-2 (pink curves, right panels), are suppressed by Fer-1 (green curves) but not by MitoTEMPO (black curves) in either A549 (top panels) or H1975 cells (bottom panels) in media not supplemented with selenium as determined 24 h after addition of compounds; four-parameter dose-response curve fit to *n* = 4 technical replicates; B) A549 cells (left panels), H1975 cells (middle panels) and HT1080 cells (right panels) grown with (black bars) or without (grey bars) 100 nM selenium supplementation in the growth medium were treated for 24 h as indicated whereupon viability was determined, here shown as percent of DMSO-treated controls. The additional treatments are a known inhibitor of ferroptosis (Ferrostatin-1), a strong antioxidant (NDGA), iron chelators (DFO and DPD50), an inhibitor of necrosis (Necrostatin-1), or a caspase inhibitor/apoptosis blocker (z-VAD-fmk). Data from *n* = 4 technical replicates are shown. Unpaired, two-tailed *t*-test; *P < 0.05, **P < 0.01, ***P < 0.001, ****P < 0.0001 in comparison to DMSO control of same Se condition; non-significant differences are not noted. (For interpretation of the references to colour in this figure legend, the reader is referred to the Web version of this article.)

### 2.4 Cytotoxicity induced by RSL3, but not by other TXNRD1 inhibitors, can be rescued by ferrostatin-1

Having found that RSL3 inhibits TXNRD1 with similar efficacy as the previously developed TRi-1 and TRi-2 compounds [17], we next compared these three compounds with regards to their efficacies in eliciting cell death, asking whether all three compounds would display similar ferroptotic features in the cell death that they trigger. First, we compared the cytotoxic effects of the three compounds in lung adenocarcinoma A549 cells, which have an unusually high activity and expression of TXNRD1 that is also known to be able to modulate the cytotoxic efficacy of different anticancer drugs [34,35]. Additional expression information for the cell lines used can be found in Extended Data Table 1. We performed this study without specific selenium supplementation because additional selenium in the medium can blunt the cytotoxicity of RSL3 (see below), possibly by augmenting GPX4 expression more than increasing the TXNRD1 levels. This first analysis revealed that, as expected based upon literature findings, RSL3 was less potent towards A549 cells (IC_50_ around 0.5 μM) compared to what is seen with more RSL3-susceptible cell lines, and that the cell death triggered by RSL3 was suppressed at least 20-fold by addition of ferrostatin-1, a key feature of ferroptosis, but not by the mitochondrial antioxidant MitoTEMPO (Fig. 3A, left panels). The cytotoxicity of TRi-1 or TRi-2 in A549 cells was less potent when compared to RSL3 (IC_50_ values of about 20 μM for TRi-1 and 5 μM for TRi-2), but co-treatment with neither ferrostatin-1 nor MitoTEMPO had any effect (Fig. 3A, top middle and top right panels). When these experiments were performed with H1975 human non-small cell lung cancer cells, which are more susceptible to RSL3, the cytotoxicity profiles were very similar to the findings with A549 cells, but with a lower IC_50_ value of 150 nM for RSL3, and slightly decreased values for the TRi compounds (Fig. 2A, bottom panels). These initial results suggested that RSL3 triggers a typical ferroptotic cell death, as expected, while TRi-1 and TRi-2 do not.

### 2.5 The cytotoxicity profiles of RSL3 and other TXNRD1 inhibitors differ

To further compare the cytotoxicity profile of the compounds, we next assessed whether selenium supplementation or the inclusion of other cell death pathway blockers could reveal any further details in differences in cell death mechanisms as triggered by RSL3 compared to that triggered by the two TRi compounds. Selenium supplementation revealed a dramatic impact on the effects of RSL3 towards A549 cells. At 24 h, non-selenium supplemented A549 cell viability was reduced to 10% with 5 μM RSL3 treatment compared to DMSO controls (Fig. 3B, top left panel; RSL3 activity shown with dashed pink line). However, if the cells had been supplemented with 100 nM sodium selenite, the same treatment resulted in a maintained viability of approximately 78%. (Fig. 3B, top left panel; indicated with green upward arrow). In A549 cells without selenium supplementation treated with RSL3, the cell death displayed clear signs of ferroptosis, as it was blocked by ferrostatin, NDGA (another strong antioxidant), or the iron chelators DFO and DPD50 (Fig. 3B, top left panel; indicated by blue box). RSL3-treated A549 cells in absence of selenium supplementation were also partially protected by nectrostatin, suggesting a component of necrosis in addition to ferroptosis, which has also been shown by others in hepatocellular carcinoma [36]. As expected, the apoptosis-blocking caspase inhibitor z-VAD-fmk did not protect these cells from RSL3, again compatible with the definition of ferroptotic cell death. The effects of TRi-1 and TRi-2 were in stark contrast to the cytotoxicity profile of RSL3. Neither selenium supplementation nor addition of any of the blocking compounds had any effect on the cytotoxicity of the TRi compounds. With TRi-1, treatment under conditions resulting in a cell viability of about 40%; this was seen under all the tested conditions (Fig. 3B, middle row left panel; pink dashed line). With TRi-2, the effect was similar; however, TRi-2 displayed stronger toxicity towards the selenium supplemented A549 cells as compared to those that had not been supplemented (Fig. 3B, bottom left panel; indicated by downward green arrow). Analyzing the effects with two cell lines used to study ferroptosis, H1975 and HT1080 cells, both of which have lower TXNRD1 activity and expression compared to A549 cells [35], similar profiles in cytotoxicity were seen with all three compounds as had been observed with the A549 cells. These cell lines, however, showed differential responses to selenium supplementation with regards to the effects of RSL3 treatment; a partial rescue was seen in H1975 cells, but no rescue in HT1080 (Fig. 3B, top middle and top right panels). This may be partially explained by a differential expression of GPX4 in these two cell lines: HT1080 cells have significantly lower GPX4 levels, while H1975 cells have a much higher expression of GPX4 (Extended Data Table 1) [35]. TRi-2 exhibited a moderate increase in cytotoxicity in the H1975 cell line with selenium supplementation, but not in HT1080. TRi-1 showed no stark difference in response profiles in any of the three cell lines.

### 2.6 FINs, but not other TXNRD1 inhibitors, modulate GPX4 migration

**Fig. 4:**
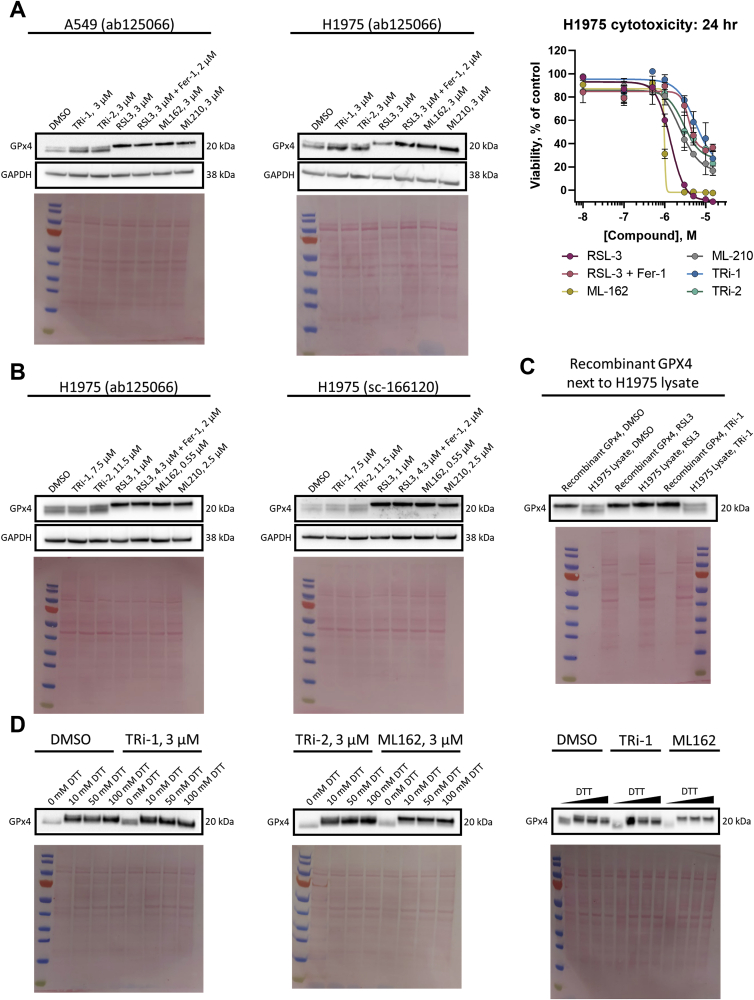
**FINs, but not TRi compounds, affect GPX4 migration.** A) A549 (left panel) or H1975 cells (middle panel) were seeded 18 h (with or without 2 μM Ferrostatin-1 pre-treatment for 14 h) before being treated with 3 μM of compound for 24 h. Proteins were resolved by reducing SDS-PAGE (10 mM DTT) and probed with the ab125066 anti-GPX4 antibody. GAPDH and Ponceau stains acted as the loading control; blots are representative of *n* = 3 (left panel), or *n* = 2 (middle panel) biological replicates. The 24-hour cytotoxicity profiles of the compounds in H1975 cells under these conditions are also presented (right panel). Cytotoxicity data are presented as mean ± s.d. of the average of *n* = 3 technical replicates; B) H1975 cells were seeded 18 h (with or without 2 μM Ferrostatin-1 pre-treatment for 14 h) before being treated with ∼IC_50_ concentrations of compound for 6 h. Proteins were resolved by reducing SDS-PAGE (10 mM DTT) and probed with either ab125066 anti-GPX4 antibody (left panel), or sc-166120 anti-GPX4 antibody (right panel). GAPDH and Ponceau stains acted as the loading control; blots are representative of *n* = 1 (right) biological replicate; C) Recombinant GPX4 was incubated with DMSO, 8 μM RSL3 or 0.5 μM TRi-1 for 30 min (right panel) in presence of full reaction mixture. H1975 lysates were prepared as in A. Proteins were resolved by reducing SDS-PAGE (10 mM DTT) and probed with the ab125066 anti-GPX4 antibody. Ponceau staining was the loading control; blot is representative of n = 2 technical replicates D) A549 cells were seeded 18 h before being treated with 3 μM compound for 24 h. Together, the left and central panel were from the same biological replicate, the right panel from a separate replicate. Proteins were resolved by SDS-PAGE with samples pre-treated with varying concentrations of DTT (0–10 mM in right panel, or otherwise indicated) and probed with the ab125066 anti-GPX4 antibody. Note the sharper slower migrating GPX4-immunoreactive band in samples from cells treated with FINs but not TRi compounds. n = 2 biological replicates from A549 (A, left) were used, left and middle blots from the same replicate, and right from a second.

Some reports show that RSL3, and other FINs, at least partially, modulate the expression levels of GPX4 as a means of its inhibition. We thus performed immunoblot analyses for GPX4 in A549 cells, finding no obvious changes in its expression levels compared to DMSO controls after treatment with 3 μM of TRi-1 or TRi-2 for 24 h. However, we noted a double band with GPX4 immunoreactivity in these analyses and, specifically upon treatment with the same 3 μM dose of the FINs, the faster migrating band disappeared and instead a more prominent upper band was seen (Fig. 4A, left panel). This effect was not modulated by the addition of 2 μM Ferrostatin-1, and it was a consistent finding in H1975 cells both at 24 h (Fig. 4A, middle panel) and when using IC_50_ values, as determined by cell viability assays at 24 h (Fig. 4A, right panel), with treatment for 6 h (Fig. 4B). We also saw the same results with another antibody against GPX4, which speaks against unspecific bands (Fig. 4B, right panel). When the same samples were analyzed next to pure recombinant GPX4 enzyme, it seems clear that the slower migrating upper band corresponds to the intact selenoprotein (Fig. 4C). Thus, the faster migrating band could represent some yet unidentified cellular modification of GPX4, found in DMSO controls and TRi compound treated cells, which specifically disappears upon treatment of the cells with FINs. Reasoning that the redox state of GPX4 somehow might be involved we also analyzed samples in the absence of reducing agent (DTT) or with higher concentrations of DTT, and, indeed, the GPX4 immunoreactive band migrated faster in the absence of DTT, irrespective of compound treatment (Fig. 4D). It is however not certain that the difference between the two GPX4 bands seen in the cell lysates (Fig. 4A and B) could be explained by the redox state, especially since DTT was included in the samples of those analyses, and the GPX4 band in the absence of DTT migrates even faster than the fast-migrating band in DMSO treated DTT-reduced cell lysates. We can solely conclude here that the FINs, but not the TRi compounds, seem to have affected GPX4 migration in the gels as compared to that seen in cell lysates from DMSO controls.

## 3 Discussion

Our findings have revealed that none of the three class II FINs, RSL3, ML162, and ML210, are direct inhibitors of GPX4. Instead, we found that RSL3 and ML162 are direct and potent inhibitors of TXNRD1, another cytosolic selenoprotein that propels an array of antioxidant thioredoxin-dependent enzymes and regulatory redox pathways in cells, but which has typically not yet been considered in relation to ferroptosis. While RSL3 and ML162 showed similarly potent inhibition of TXNRD1 as the previously characterized TXNRD1 inhibitors, TRi-1 and TRi-2, the TRi compounds and the FINs showed highly divergent cytotoxicity profiles. As expected, the FINs triggered a ferroptotic cell death that could be blocked by ferrostatin-1 and iron chelators, to varying degrees, but not by caspase inhibition. In contrast, TRi-1 and TRi-2 triggered a cell death that could not be rescued by any of the tested cell death pathway inhibitors. Though the importance of understanding the implications of TXNRD1 inhibition by FINs in the context of ferroptosis should not be overlooked, it is possibly more curious that the cell death connected with this inhibition does not mimic that triggered by the TRi compounds. These compounds are the most specific TXNRD1 inhibitors yet described [17,[20], [21], [22], [23]]. Treatment with the potent but promiscuous TXNRD1 inhibitor, auranofin, in a hemochromatosis mouse model showed an increase in hepatic malondialdehyde (MDA), a product of lipid peroxidation, and Ptgs2 mRNA levels, which is highly associated with ferroptosis [37]. Those effects were reversed with co-treatment of Ferrostatin-1. However, auranofin has also been shown to decrease GPX4 expression levels, possibly suggesting a more classic route to these ferroptosis characteristics [22]. Interestingly, TRi-1 treatment in C57BL/6 mice was shown by others to increase MDA and Ptgs2 levels similarly to auranofin, an effect also rescued with Fer-1 treatment, suggesting ferroptosis being triggered by TRi-1 in that study [37], in contrast to our findings herein. Perhaps the triggered cell death pathways are context dependent and yet unknown orthogonal off-target effects can differ between FINs and TRi compounds, which could explain their resulting cytotoxicity profiles. However, based upon our present findings, prior studies using RSL3 and ML162 clearly need to be reevaluated knowing that TXNRD1 is potently and directly inhibited by these compounds, rather than GPX4 as has previously been assumed. Additional studies parsing the molecular mechanisms of cell death by FINs as well as TRi compounds are required with a focus on the possible direct and indirect involvement of TXNRD1 as well as GPX4.

We could not detect any direct inhibition of either TXNRD1 or GPX4 by ML210 and recent studies have shown that ML210 requires a cellular context to enact its effects that lead to ferroptosis, with cellular metabolism of the compound proposed to yield metabolites that inhibit GPX4 [12]. Surprisingly, that paper also identified TXNRD1 as a major hit in a ML210 genome-wide CRISPR suppressor screens [12], while TXNRD1 did not show up as a hit in the RSL3 screen. It is interesting that we here detected an initial increase in RX1 signal upon treatment with ML210 and suppression at higher concentrations, suggesting such metabolites may also be involved in modulating TXNRD1 activity. A previous study found, similarly to us, that RSL3 does not directly inhibit pure GPX4, but its inhibition was seen in presence of the adaptor protein 14-3-3ε, and interestingly only when it was present in its reduced state [11]. Though, a proteomics analysis using RSL3 identified direct binding to the Sec residues of both GPX4 and TXNRD1, as well as a third selenoprotein, SELK [38]. Two earlier studies furthermore showed that the NADPH-driven TXNRD1/TXN1 system can support GPX enzymatic activity in the absence of GSH [39,40]. It should finally be noted that an unbiased proteomics-based screen for cellular TXNRD1 substrates also identified both GPX1 and GPX4 as likely downstream substrates of the enzyme [41]. Thus, TXNRD1 and GPX4 targeting may indeed be functionally and directly linked in a cellular context. It is however noteworthy that the FINs, but not the TRi compounds, were here found to affect migration of the GPX4 immunoreactive band in SDS-PAGE analyses, which was an effect not modulated by treatment with ferrostatin-1 and thus not directly related to the ferroptotic cell death. Though this may be expected as there was no major effect previously seen with TRi-1 or TRi-2 treatment on GPX4 thermostability [21]. The findings thereby suggest that the FINs could specifically modulate GPX4 integrity in a manner not seen with the TRi compounds, although all compounds in our analysis were used at concentrations inhibiting TXNRD1 to a similar extent (and, again, none of them inhibiting GPX4 in direct enzyme assays). The molecular mechanisms behind these phenomena and their potential importance in relation to ferroptosis should be studied further.

The potent cytotoxicity of TRi compounds as found here could suggest that they might be highly toxic *in vivo* and towards healthy cells, but that does not seem to be the case. Both TRi-1 and TRi-2 when given at high doses to mice lack overt toxicity, although they still display antitumoral properties [17]. Such lack of overt toxicity to mice (and human) despite a potent cytotoxicity in cancer cell cultures is also seen with auranofin, which is highly toxic towards tumor cells in culture but safe for human use; auranofin (Ridaura®) has long been used to treat arthritis and is currently in several clinical trials for evaluation as anticancer therapy (see www.clinicaltrials.gov). In connection with forthcoming assessments of the possible effects of different TXNRD1 inhibitors in relation to ferroptosis, useful analyses would be to compare the proteomics and transcriptomics profiles in cells treated with either RSL3, ML162, TRi-1 or TRi-2, with the aim of identifying any cellular effects that could explain why the FINs trigger typical ferroptosis while TRi-1 and TRi-2 do not, although all these four compounds are evidently similarly potent TXNRD1 inhibitors.

## Funding

This work was supported by the Intramural Research Program of the National Institutes of Health National Center for Advancing Translational Sciences. DMC was supported through a joint NIH-Karolinska Insitutet graduate training program. ESJA acknowledges funding from Karolinska Institutet, The Knut and Alice Wallenberg Foundations (KAW 2019.0059), The Swedish Cancer Society (21 1463 Pj), The Swedish Research Council (2021–02214), National Laboratories Excellence program under the National Tumor Biology Laboratory project (2022–2.1.1-NL-2022-00010) and the Hungarian Thematic Excellence Programme (TKP2021-EGA-44) and The National Research, Development, and Innovation Office (NKFIH) grant ED_18-1-2019-0025.

## Author contributions

DMC, MDH, and ESJA conceived the study and designed the experiments. DMC, CH, KCS, MHR, and RG performed the experiments. QC produced recombinant proteins, including selenoprotein GPXs and TXNRDs. DMC and ESJA wrote the final manuscript, which was revised by CH, KCS, MHR, RG, QC, and MDH.

## Methods

Recombinant human proteins—Recombinant human selenoprotein GPX and TXNRD isoenzymes as well as recombinant human GR were cloned, expressed, and purified as described previously [[26], [27], [28]].

Biochemical assays—GPX assays were run as previously described [[26], [27], [28]] in a clear medium-binding 96-well SpectraPlates (PerkinElmer). Briefly, 198 μL of 10 nM GPX1 or 200 nM GPX4 were incubated with 2 μL compounds or DMSO in TES buffer (50 mM Tris-HCl, 2 mM EDTA (pH 7.5), 150 mM NaCl) with BSA at room temperature for 30 min. Then, 40 μL of master mix (100 nM GR, 1 mM GSH, 0.5 mM NADPH in TES buffer) was added. To start the reaction, 10 μL of Cumene hydroperoxide ([0.5 mM] in 50% EtOH) was then added. Absorbance at 340 nm was measured using a Tecan Infinite® M Nano microplate reader every 20 s for 30 min. Four replicates were run, unless otherwise stated.

GR counter assay was run by incubating 198 μL of GR and BSA in TES buffer with 2 μL compound for 30 min before dispensing 50 μL GSSG and NADPH in TES for final concentrations of 100 nM GR, 1 mM GSSG, 0.4 mM NADPH, 0.01% BSA. Absorbance at 340 was measured as above.

TXNRD1 selenite and DTNB assays were run as previously described [[26], [27], [28]]. Dose-response curves and IC_50_s were generated using Excel and GraphPad Prism. Briefly, initial slopes of NADPH consumption were normalized to DMSO controls and IC_50_s were calculated using log(inhibitor) vs. response—Variable slope (four parameters).

Nano differential scanning fluorimetry—Real-time monitoring of fluorescence emission at 330 nm and 350 nm (excitation wavelength: 280 nm) and backscattering absorbance of GPX4 and TXNRD1 samples in the presence of compound was performed using a NanoTemper Prometheus NT.48 instrument. First, 0.25 mg/mL enzyme was incubated with 100 μM compound or DMSO for 15 min at room temperature. Standard capillaries were used, and temperature was increased from 25 to 95 °C with a ramp rate of 4.0 °C/min. Three biological replicates were carried out for each condition, and their means and standard deviations are depicted.

Cell culture—H1975 and A549 cells were cultured in RPMI-1640 medium (HyClone) with Earle's salts supplemented and penicillin/streptomycin (100 μg/ml), while HT1080 cells were cultured in MEM medium for Fig. 2 experiments. For all other cellular experiments, A549 cells were cultured in DMEM 4.5 g/L glucose medium (Gibco), and H1975 cells in RPMI-1640 ATCC Modification (Gibco). Cell cultures were maintained at 37 °C in 5% CO2 in a medium containing 10% fetal bovine serum (FBS) (GE Healthcare, A15-102 for Fig. 2 experiments and Sigma, F7524 for all others). Experiments were performed with or without supplementation with 100 nM sodium selenite as described.

Cellular TXNRD1 assay—20,000 or 10,000 A549 cells (for 4 h or 12 h and 24 h compound incubation respectively) were seeded in 96-well plates (microplates, 96 well, F-bottom, black, clear bottom, ViewPlateTM-96 F TC, PerkinElmer), in a total volume of 100 μL full DMEM (supplemented with 10% FBS and 100 nM Na2SeO3). The cells were incubated for 16 h at 37 °C and 5% CO_2_, followed by media change and addition of indicated compounds at respective concentrations (0.1 μM–20 μM), including 0.6% DMSO. The cells were subsequently incubated for the respective mentioned time, washed once with full DMEM and fresh, full DMEM, containing 75 μM RX1 and 0.75% DMSO, was added and the first fluorescent measurement was conducted immediately (timepoint = 0) using a Tecan Infinite M200 plate reader (ex/em 355/520). To follow the kinetic activation of RX1, a fluorescent measurement was done every hour for 6 h. Between measurements, cells were stored at 37 °C and 5% CO_2_. As a control, cells treated with compounds, but not with RX1, just the respective DMSO content, were used for background fluorescent. To determine how the cells were viable throughout the RX1 assay, the control cells were used for a viability measurement, using CellTiter-Glo® Luminescent Cell Viability Assay, after the final fluorescent measurement. For final data analysis, raw data was processed using Microsoft Excel and GraphPad Prism. In brief, the slope of the activated RX1 fluorescence increase was in each case calculated and normalized, using the viability measurement of the control cells.

Cytotoxicity assays— Cells were seeded in a 96-well plate at the density of 3 × 10^3^ per well and allowed to adhere overnight. After which, cells were treated with various concentrations of compound for the indicated times, whereupon cell viability was determined using Cell Quanti-Blue™ Cell Viability Assay Kits (Nordic Diagnostica, CQBL-10K). Briefly, QUANTI-Blue Reagent (10 μL) was added to each well, incubated at 37 °C in 5% CO_2_ for 3 h, and then the plates were measured at 540nm/600 nm (Ex/Em) using the Tecan Microplate Reader (Morrisville NC, USA).

Western blots—A549 and H1975 cells cultured for at least 72 h in the presence of 100 nM Na_2_SeO_3_ (Sigma) were seeded in 90 mm dishes (VWR) at 1 × 10^6^ cells per dish for 18 h prior to main treatments. Four hours after seeding dishes, cells were pretreated with 2 μM Fer-1 for 14 h prior to adding RSL3, when indicated. After 14 h, main treatments with TRi-1, TRi-2, RSL3, ML162, ML210 or 0.03% v/v DMSO in doses indicated in each figure were added for 6 or 24 h. Total DMSO content was 0.05%. Cells were then scraped, washed in PBS, and suspended in 150–200 μL lysis buffer containing 50 mM Tris-HCl (pH 7.5), 2 mM EDTA, 0.15 M NaCl, 1% Triton X-100, and cOmplete protease inhibitor cocktail (Roche). Supernatants were collected after 30 s of sonication, and 30 min centrifugation at 17000×*g*, and protein concentration was measured using Pierce bicinchoninic acid (BCA) protein assay kit (Thermo Fischer Scientific). Recombinant protein reactions were prepared essentially as the biochemical assays described above, with 250 nM GPX4 incubated with compounds or 0.5% DMSO for 30 min in the presence of 0.25 mM NADPH and 0.25 mM cumene hydroperoxide. Cell lysates or recombinant GPX4 reactions (20–25 μg total protein and 15 ng recombinant GPX4) denatured at 95 °C in LDS buffer (Novex Life Technologies) containing 10 mM DTT (or DTT as indicated in figure legends) were loaded onto Bolt Plus 4–12% BisTris precast gels (Thermo Fischer Scientific), separated by SDS-PAGE at 165V for 40 min and dry transferred to nitrocellulose membranes (iBlot 2) at 20 V for 7 min. Membranes, after blocking in 5% milk, were probed for GPX4 (EPNCIR144, ab125066, LOT GR3438547-5, Abcam) and, where indicated, GPX4 (B-12, sc-166120, LOT B0519, Santa Cruz Biotechnology), and GAPDH (0411, sc-47724, LOT A0622, Santa Cruz Biotechnology), all dissolved in PBST with 1% BSA. Chemiluminescent signal was detected using anti-rabbit and anti-mouse HRP-IgG secondary antibodies (Southern Biotech, 4030–05, LOT A2718-MM00 and 1030–05, LOT L3919-XE81B respectively) and ECL detection reagent (Cytiva Amersham). Protein quantification based on band densitometry was performed in Quantity One and Image Lab (BioRad).

Statistics— statistical significance was assessed in GraphPad Prism using Student's t-test (two-tailed distribution, two-sample, unequal variance).

## Declaration of competing interest

ESJA has patents on TRi compounds for development towards cancer treatment. ESJA and QC are shareholders in Selenozyme AB, a company selling recombinant selenoproteins.

## Data Availability

Data will be made available on request.
