# Supplementary material for: The ferroptosis inducing compounds RSL3 and ML162 are not direct inhibitors of GPX4 but of TXNRD1
Source: Redox Biol. 2023 Apr 17;62:102703. doi: 10.1016/j.redox.2023.102703 (PMC10149367; doi:10.1016/j.redox.2023.102703)
Supplement: Multimedia component 1 [file mmc1.pdf]

The ferroptosis inducing compounds RSL3 and ML162 are not direct inhibitors of GPX4 but of TXNRD1

Dorian M. Cheff,[1,2] Chuying Huang,[1,&] Karoline C. Scholzen,[1] Radosveta Gencheva,[1] Michael H. Ronzetti,[2] Qing Cheng,[1] Matthew D. Hall,[2] Elias S.J. Arnér,[1,3,#]

[1]Division of Biochemistry, Department of Medical Biochemistry and Biophysics, Karolinska Institutet, SE-171 77 Stockholm, Sweden.

[2]Early Translation Branch, National Center for Advancing Translational Sciences, National Institutes of Health, Rockville, Maryland 20850, United States.

[3]Department of Selenoprotein Research and the National Tumor Biology Laboratory, National Institute of Oncology, Budapest, Hungary

[&]Present address: Department of Medical Oncology, Enshi Tujia and Miao Autonomous Prefecture Central Hospital, Hubei Province, China.

[#]Corresponding author. E-mail: elias.arner@ki.se

Results

We could not detect GPX4 inhibition by RSL3 at any concentration up to 80 µM (**Extended Data Fig. 2A, left panel**). Measuring inhibition of TXNRD1 activity, as a complement to the analyses shown in the main text of this article, we also assessed this using two orthogonal approaches: 1. by the measurement of NADPH consumption coupled to direct selenite reduction of TXNRD1, either through the decrease in absorbance at 340 nm or decrease in its fluorescence, and 2. measuring TXNRD1-catalyzed DTNB reduction based upon quantification of TNB^- anions absorbing at 412 nm as formed upon TXNRD1-catalyzed reduction of the disulfide in DTNB. Using these assays, we could confirm that the RSL3 inhibition of TXNRD1 was not an artifact due to specific assay conditions, with both RSL3 and ML162 showing complete inhibition of TXNRD1 at the higher end of the concentrations assessed here (2.8 ± 0.9% and 5.8 ± 0.9% remaining activity with just 5 µM of RSL3 and ML162, respectively), and with curve-fit IC_{50} values of 745.1 nM for RSL3 and 515.4 nM ML162 (**Extended Data Fig. 2A, middle and right panels, and 2B**). ML210 did not show any inhibition of TXNRD1 in any of the assays.

The mitochondrial TXNRD2 isoenzyme was also assessed, towards which both RSL3 and ML162 showed inhibitory activity, but to a lesser extent than with TXNRD1. At the top concentration, RSL3 inhibited TXNRD2 down to 36.2 ± 8.6% activity, and ML162 to 35.1 ± 1.3% activity. The IC_{50} values for TXNRD2 under these assay conditions were 9.2 µM and 4.0 µM, respectively (**Extended Data Fig. 2B**). Like with TRi-1 and TRi-2, inhibition of both TXNRD1 and TXNRD2 by RSL3 and ML162 was essentially dependent on pre-reduction of the enzyme by NADPH in order to yield potent and complete inactivation. Both compounds completely lost inhibitory activity against TXNRD2 if the enzyme was not pre-reduced with NADPH, while TXNRD1 displayed some inhibition also when incubated first with RSL3 and ML162 in the absence of NADPH and subsequently assayed for activity; however, this could also be due to carry-over of the compounds to the NADPH dependent enzyme activity assay. Because the reduction step with NADPH reduces a selenenylsulfide at the C-terminal active site of the oxidized state of the enzyme to a selenolthiol, these results suggest that RSL3 and ML162 mainly inhibited the two TXNRD isoenzymes by electrophilic attack and derivatization of the selenolate at the C-terminal active site of the NADPH-reduced enzyme. That some inhibition of oxidized (non-reduced) TXNRD1 may occur could however suggest that the compounds might also bind some other additional site(s) in the enzyme. That notion is further strengthened by the fact that thermostability of TXNRD1 was also affected at higher concentrations of compounds when incubated with the enzyme in the absence of NADPH (see main text).

Intrigued by the reproducible effect on the GPX4 band with FIN treatment (see main article text), we also ran the H1975 samples with a different GPX4 antibody (sc-166570, mouse monoclonal), to make sure our double-band as detected was not unspecific due to the antibody. This gave the same trend wherein the FINs resulted in one distinct band, while the DMSO control and treatment with either of the TRi compounds in addition displayed a second, faster moving band (**Fig. 4B, right panel**).

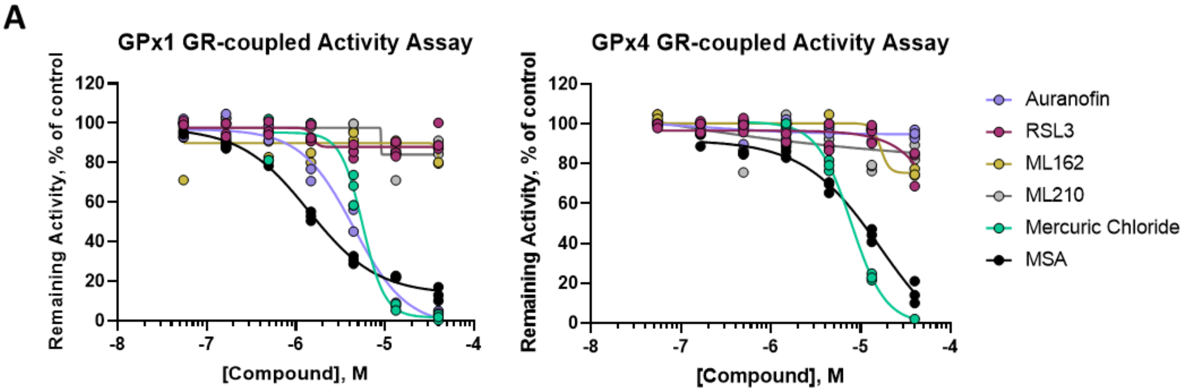

Extended Data Figure 1: Inhibition of GPX1 and GPX4, or lack thereof, by prior art compounds. Activities of GPX1 (left panel) and GPX4 (right panel) were determined in the presence of increasing concentrations of selected compounds (as indicated in the figures) having been considered or reported in the literature as inhibitors of GPX4; Four-parameter dose-response curve fit to the average of *n* = 3 technical replicates.

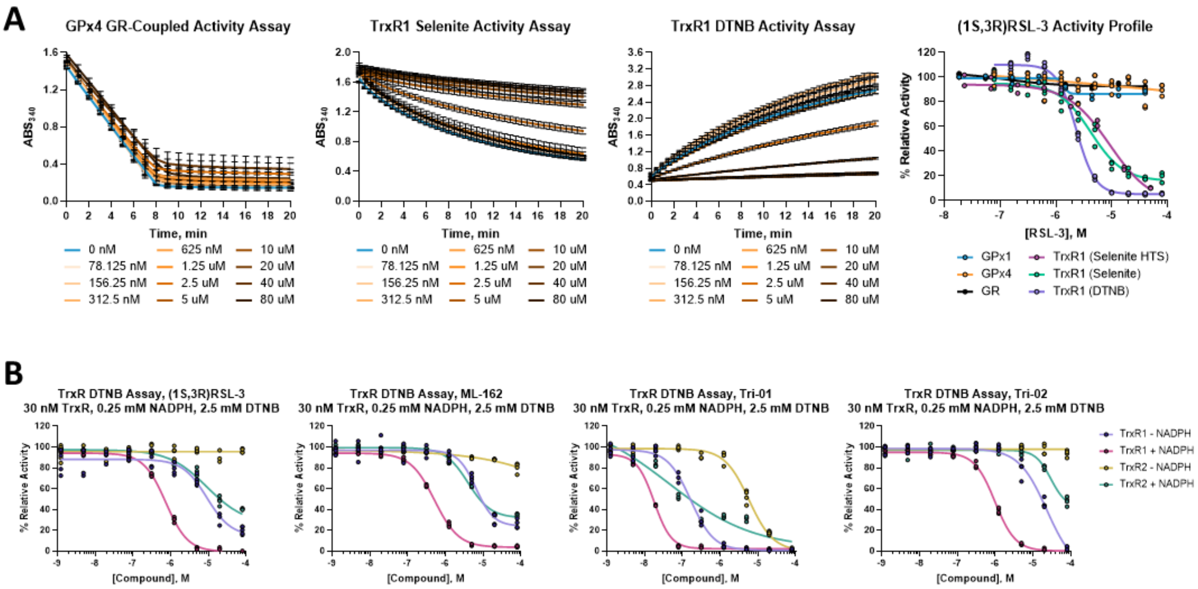

Extended Data Figure 2: A) RSL3 does not inhibit GPX4 at any dose points (left panel) but shows dose-dependent inhibition of TXNRD1 in both a selenite reduction assay in both 96-well (middle left panel) and 1536-well format (not shown), as well as a DTNB assay (middle right panel). RSL3 did not inhibit GPX1 or GR (right panel); B) Inhibition of TXNRD1 and TXNRD2 by RSL3 (left panel), ML162 (middle left panel), TRi-1 (middle right panel), and TRi-2 (right panel) are modulated by NADPH pre-treatment; Data are presented as mean ± s.d.; Four-parameter dose-response curve fit to the average of n = 3 technical replicates (n = 2 for TrxR1 Selenite HTS).

	**Extended Data Table 1: Gene expression of related targets**		
**GENE ID**	**A549**	**HT1080**	**H1975**
TXNRD1	2.08	0.27	0.24
TXN	0.75	0.46	0.62
GPX4	-0.71	-1.82	2.18
AIFM2/FSP1	0.98	-0.16	-0.07
SLC7A11	1.69	-0.05	-1.01
GCLC	2.32	-1.00	-0.35
GCLM	2.74	0.38	-0.16

Extended Data Table 1: Gene expression of related targets for cell lines used. Data is reported as Z-score for each cell line above or below the population mean of all CCLE cell lines tested in RNAseq analysis conducted by the Broad Institute (https://depmap.org/portal/) (35).

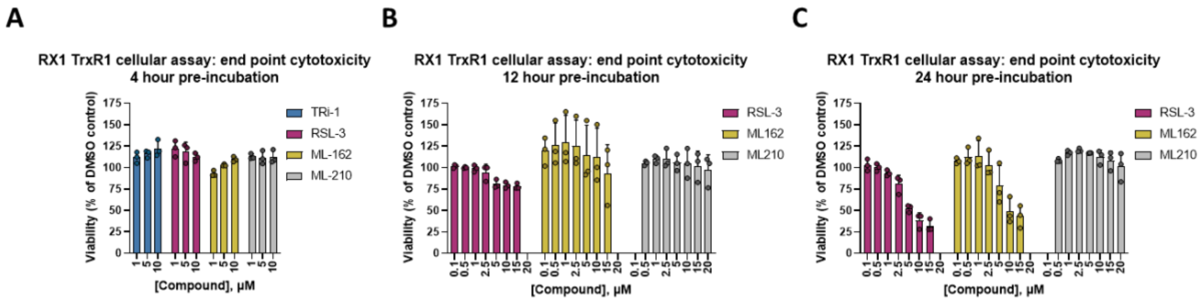

Extended Data Figure 3: A549 cytotoxicity in cellular TXNRD1 experiments following final RX1 measurement (as shown in **Fig. 2**). A) Cell viability following 4-hour incubation with compounds; B) Viability following 12-hour incubation with compounds; C) Viability following 24-hour incubation with compounds. The cell viability in was in each case measured using the Cell Quanti-Blue™ Cell Viability Assay Kit and the data are presented as mean ± s.d. of n = 3 biological replicates, each containing n = 3 technical replicates.
